# Better perceived health among the Swedish-speaking minority as compared with the Finnish-speaking majority in Finland: a cross-sectional study with an intergenerational perspective

**DOI:** 10.1177/14034948241258674

**Published:** 2024-07-31

**Authors:** Sakari Suominen, Diana Stark Ekman, Jan Saarela, Salla-Maarit Volanen, Säde Stenlund, Lauri Sillanmäki, Markku Sumanen

**Affiliations:** 1 University of Skövde, School of Health Sciences, Sweden; 2 Turku University Hospital, Research Services, Finland; 3 Department of Public Health, University of Turku, Finland; 4 Social Sciences, Faculty of Education and Welfare Studies, Åbo Akademi University, Vasa, Finland; 5 Folkhälsan Research Centre, Helsinki, Finland; 6 Faculty of Medicine and Health Technology, University of Tampere, Finland

**Keywords:** Cultural minority, ethnic minority, perceived health, Swedish speaking Finns, comparative study, cross-sectional study, intergenerational

## Abstract

**Background:**

Previous research has shown that the Swedish speaking minority in Finland has slightly but significantly better health compared with the Finnish speaking majority. However, a clear explanation for this is lacking.

**Aim:**

The aim of the study was to explore differences of perceived health comparing three groups: Swedish speakers with reported dominance of Swedish also in the preceding generation; contemporary Finnish speakers with reported dominance of Finnish in the preceding generation and a group with a reported mixed-language structure of Finnish and Swedish between generations.

**Individuals and methods:**

Health and Social Support is an on-going population-based survey initiated in 1998 (*N* = 64,797), aimed at working-age adults. The present study is based on the 2012 follow-up survey, which included a question on the dominating language (Swedish or Finnish) of the respondents and their parents. The outcome was perceived health, which in this study was dichotomized to very good/good and intermediate/poor/very poor. The statistical analysis was carried with logistic regression, using SAS software. Age, gender and occupational training were included as covariates in the multivariable analysis.

**Results:**

This study found that the Swedish-speaking group in Finland report better perceived health compared with the Finnish-speaking group (odds ratio 1.28, 95% confidence interval 1.04–1.57, *p* < 0.001). The health of the mixed language-speaking group fell between the other two groups.

**Conclusions:**

**The results gave some support to a culturally mediated mechanism for the health advantage of Swedish speakers. Cultural features of Swedish-speaking groups in Finland may also support health promotion of the Finnish-speaking majority.**

## Introduction

The Swedish-speaking minority in Finland (currently 5% of the total population [1]) has demonstrated a small noticeably better level of health compared with the Finnish-speaking majority. These differences have been noted over multiple decades, demonstrated for instance by a doctoral thesis in 1951, where slightly lower relative mortality rates [2] for Swedish speakers were found. This difference in mortality risks has been shown over many years [3–8]. The age-standardized mortality rate ratio between Swedish- and Finnish-speaking men aged 18–50 years is about 0.60 and between Swedish- and Finnish-speaking women about 0.70 [9], with these differences only partially attributed to socio-economic and demographic factors.

Swedish-speaking workers in Finland have lower risks for needing disability pensions before retirement age, and lower levels of sickness absence, than Finnish speakers [10,11]. Several ideas have been presented to explain these observations, including differences in health behaviour [12,13], genetic variation [14] and the fact that the Swedish speakers live in regions with lower levels of morbidity [8]. Somewhat more favourable living conditions have also been proposed as potential contributors to the health advantage of Swedish speakers, although causality may be disputed [15].

In seeking an explanation for differences in health between Swedish and Finnish speakers, the long history of relative socio-economic advantage of some Swedish speakers has been proposed as a factor [16], relating back to the time Finland was part of Sweden, with a more privileged subgroup of the Swedish-speaking population living mostly in the southern and western area. However, this urban Swedish-speaking group’s socio-economic advantage has never applied to the entire population of Swedish speakers, and the over-representation of Swedish speakers among higher-paid professionals in Finland has continually decreased [17].

Use of primary languages in Finland is changing, which further complicates matters. The Swedish-speaking population of Finland has always lived concentrated on the southern and western coastline of Finland, that is, in regions that have also experienced internal migration of Finnish speakers, which may have contributed to the declining use of Swedish as a predominant language. The share of Swedish speakers in the total population has decreased from 12.9% to 5.2% during the last century, while the proportion of mixed language speakers, that is, persons with bilingual (Finnish–Swedish) background, is continually increasing [1]. Utilizing register data that link persons to both their parents, it has been shown that life expectancy of mixed language speakers lies in between that of both Swedish-speaking and Finnish-speaking groups [18]. Likewise, the standardized mortality risk of working-aged persons with mixed language background is between that of persons in Swedish-speaking and Finnish-speaking groups, and this pattern is particularly clear for alcohol-related mortality [18]. A person’s own language, their dominant language, has more explanatory power than their parents’ [18].

A new strand of research, which the present study is part of, attempts to dissect the Swedish–Finnish health differential from the perspective of more than one generation and poses the question of whether cultural pathways might also play a role. The role of the primary language beyond a strict one-generational perspective requires further attention, as language use appears to be shifting in Finland.

## Aim

The aim of the study was to explore differences of perceived health comparing three groups, which were: (1) contemporary Swedish speakers with reported dominance of Swedish in the preceding generation, (2) contemporary Finnish speakers with reported dominance of Finnish in the preceding generation and (3) a group with a reported mixed-language structure of Finnish and Swedish between the generations.

## Methods

### Study population, survey and survey content

This survey is part of a longer-running population-based postal survey in Finland, the Health and Social Support (HeSSup) study [19,20], which was conducted in 1998 (response rate 40.0%) followed by two follow-up survey waves in 2003 and 2012. In the original recruitment strategy in 1998, Swedish speakers were oversampled, representing about 10% of respondents, compared with this group being about 5% of the total population in Finland at the time. While the original survey was done via postal services, the two follow-ups could also be responded to electronically. All three waves of the survey were based on responses from participants who were included in the first wave, with a loss of 15% of original respondents in 2003, and an additional loss of 20% of original respondents in 2012.

This study focuses on data from the survey’s final wave in 2012. In this wave, respondents who were registered as Swedish speakers in the Finnish population register received a Swedish copy of the original Finnish questionnaire, which had undergone a standardized translation and back-translation procedure. Respondents were asked to define their dominating language, and corresponding languages of both parents. Multiple questions about perceived health, and health behaviours, were also asked.

#### Principal study variable

In 2012 the questionnaire survey included a question on the dominating language (Swedish or Finnish) of the respondents and their parents. Based on language use, three groups were assessed in this study: (1) those who had Swedish as the dominating language in their families both now and in the preceding generation (designated the Swedish-speaking group in this study); (2) those who had Finnish as the dominating language in their families both now and in the preceding generation (designated as the Finnish-speaking group in this study); and (3) those who had a mixed use of languages spoken in their families both now and/or in the preceding generation (designated the mixed language-speaking group in this study).

#### Outcome

Perceived health was measured by the following question: how would you describe your present state of health? Respondents selected one of five responses: (1) good; (2) rather good; (3) intermediate; (4) rather poor; and (5) poor. The question, formulated similarly in Finnish and Swedish, has been used in numerous national surveys in Finland. For the statistical analysis the outcome was dichotomized by bringing together the categories (1) and (2), that is, Good and Rather good to form one class and the rest of the categories in order to form another class.

#### Covariates

Covariates used included the respondent’s age (categorized into five-year groups). We restricted our analysis to persons aged 32–66 years, who amounted to 12,008 persons in the data. The age range accounts for aging among the respondents after the first wave was conducted, 12 years earlier, where this first group ranged in age from 20 to 54. Other covariates included educational level (primary, secondary or tertiary) and those that described health behaviour. The latter set referred to reported smoking (present smoker, former smoker, or never smoked) [21], alcohol consumption, calculated as grams per week (>175 for women and >273 for men; 22–175 for women and 22–273 for men; or <22 for women and men) [22,23]. We also included physical exercise as a covariate [24]. Physical exercise was converted into metabolic equivalent of task (MET) based on responses on the intensity and frequency of physical activity during a week. A MET value equal to or above 2, corresponding to approximately 30 min of walking per day, represented beneficial behaviour. This study categorized the MET values as (<2, or ⩾2).

### Data analysis

Observations with missing values on any of the variables used were omitted from the statistical analysis. The statistical analysis was carried out with multi-variable logistic regression, where we stepwise include variables, using the SAS software. Women and men were analysed jointly as there were no statistically significant interactions with sex. The specific models were each individually developed by stepwise addition of explanatory variables. However, the stepwise addition of variables was not based on an automatized *p*-value driven process but originated in an internal scrutiny of the research group.

### Ethics

The HeSSup study was approved by the concurrent joint Ethics Committee of the University of Turku and Turku University Hospital.

## Results

Table I and II report variable distribution by language group. We see that a higher share of the Swedish-speaking group was more likely to report good perceived health (81.2%) compared with those in the Finnish-speaking group (74.5%), while those in the mixed-language group lie in between the two other groups (78.1%). Compared with the Finnish-speaking group, the corresponding Swedish speakers are on average somewhat older, have more education and demonstrate somewhat more favourable health behaviour overall.

**Table I.** Straight distributions of covariates according to each language group (*n*, %).

Language group	Finnish-speaking		Mixed-language		Swedish-speaking		Total		
	*n*	%	*n*	%	*n*	%	*n*	%	*p* ^a^
Sex									0.718
Women	6833	62.8	184	63.0	514	61.4	7351	62.1	
Men	4046	37.2	108	37.0	323	38.6	4477	37.9	
Age									0.351
62–66	3341	30.7	92	31.5	285	34.1	3718	31.0	
52–56	2882	26.5	79	27.1	222	26.5	3183	26.5	
42–46	2291	21.1	65	22.3	171	20.4	2527	21.0	
32–36	2365	21.7	56	19.2	159	19.0	2580	21.5	
Vocational training									<0.001
No specific training	2104	19.4	48	16.6	136	16.4	2288	19.2	
Institute	5230	48.3	136	46.9	329	39.6	5695	47.7	
University	3484	32.2	106	36.6	366	44.0	3956	33.1	
Smoking									0.117
Present smoker	1399	13.9	50	18.6	98	13.6	1547	26.5	
Former smoker	3402	33.8	89	33.1	226	31.3	3717	63.6	
Never smoked	5262	52.3	130	48.3	398	55.1	5790	9.9	
Weekly alcohol consumption (g)									0.006
Women >175	246	2.5	6	2.4	12	1.6	264	2.4	
Men >273	283	2.8	10	3.9	14	1.8	307	2.8	
Women 22–175	3106	31.3	80	31.5	266	35.0	3452	31.5	
Men 22–273	2571	25.9	67	26.4	212	27.9	2850	26.0	
0–<22	3725	37.5	91	35.8	256	33.7	4072	37.2	
Physical exercise (MET)									0.632
<2	3057	28.3	78	26.7	222	27.0	3357	28.2	
⩾2	7753	71.7	214	73.3	600	73.0	8567	71.8	

a Pearson’s χ^2^ test for association between the covariate and the language group.

MET: metabolic equivalent of task.

**Table II.** Straight distribution of the outcome variable (perceived health) according to each language group (*n*, %).

Language group	Finnish-speaking		Mixed-language		Swedish-speaking		Total		
	*n*	%	*n*	%	*n*	%	*n*	%	*p* ^a^
Perceived health									<0.001
Good/rather good	8058	74.5	228	78.1	673	81.2	8959	75.1	
Intermediate/poor/very poor	2752	25.5	64	21.9	156	18.8	2972	24.9	

a Pearson’s χ^2^ test for association between the outcome variable and language group.

The raw difference in ‘good’ versus ‘poor’ perceived health between the Swedish-speaking group and the Finnish-speaking group corresponds to an odds ratio of 1.47 (95% confidence interval (CI): 1.23–1.77), and that between the mixed language-group and the Finnish-speaking group to an odds ratio of 1.22 (95% CI: 0.92–1.61) (Table III). These odds ratios are reduced to 1.28 (95% CI: 1.04–1.57) and 1.17 (95% CI: 0.86–1.59), respectively, when all covariates have been included.

**Table III.** Univariate and multivariable logistic regression model of perceived health (very good/good versus intermediate/poor/very poor) among Finnish and Swedish speakers in Finland adjusted for covariates, that is, potential confounders. Findings from the Health and Social Support survey. Results begin with univariate model followed by multivariable models. The results are given in odds ratios (OR) and their 95% confidence intervals (CIs).

	Model #1 *n* = 11,931		Model #2 *n* = 11,931		Model #3 *n* = 11,864		Model #4 *n* = 10,937		Model #5 *n* = 10,904		Model #6 *n* = 10,846	
	OR	95% CI	OR	95% CI	OR	95% CI	OR	95% CI	OR	95% CI	OR	95% CI
Language group												
*p*^a^	<0.001		<0.001		<0.001		0.0096		0.0224		0.0397	
Finnish-speaking	Ref.		Ref.		Ref.		Ref.		Ref.		Ref.	
Mixed	1.22	0.92–1.61	1.25	0.94–1.67	1.17	0.88–1.56	1.22	0.90–1.65	1.21	0.89–1.64	1.17	0.86–1.59
Swedish-speaking	1.47	1.23–1.77	1.56	1.30–1.87	1.41	1.17–1.70	1.33	1.09–1.62	1.29	1.06–1.57	1.28	1.04–1.57
Age												
62–66			Ref.		Ref.		Ref.		Ref.		Ref.	
52–56			1.19	1.07–1.31	1.07	0.96–1.18	1.13	1.01–1.26	2.87	2.46–3.34	1.12	1.00–1.26
42–46			2.50	2.16–2.77	2.02	1.78–2.29	2.19	1.91–2.50	2.20	1.92–2.52	2.07	1.82–2.39
32–36			3.76	3.28–4.31	2.58	2.23–2.98	2.82	2.42–3.28	2.87	2.46–3.34	2.71	2.32–3.16
Vocational training												
No specific training					Ref.		Ref.		Ref.		Ref.	
Institute					1.70	1.53–1.89	1.64	1.47–1.83	1.62	1.44–1.81	1.59	1.41–1.78
University					3.16	2.78–3.60	2.81	2.45–3.23	2.73	2.37–3.13	2.59	2.24–2.98
Smoking												
Present smoker							Ref.		Ref.		Ref.	
Former smoker							1.42	1.24–1.62	1.37	1.20–1.57	1.25	1.09–1.44
Never smoked							1.75	1.53–2.00	1.72	1.51–1.97	1.57	1.37–1.80
Weekly alcohol consumption (g)												
Women >175 Men > 273									Ref.		Ref.	
Women 22–175 Men 22–273									1.83	1.52–2.20	1.75	1.45–2.11
0–<22									1.42	1.17–1.71	1.39	1.14–1.69
Physical exercise (MET)												
<2											Ref.	
⩾2											2.28	2.08–2.52

a Multiple binary logistic regression analysis.

Ref.: reference level; MET: metabolic equivalent of task.

Estimates for effects of the covariates are very much as expected and will not be discussed at length. The odds of good perceived health are higher for younger people than for older ones, for higher educated than for lower educated, and for those with more beneficial health behaviour in terms of less smoking, less alcohol consumption and more physical exercise.

## Discussion

This study, based on a large sample of the Finnish working-age population, shows that Swedish speakers in Finland report better perceived health as compared with Finnish speakers, even when we adjust for age, education, smoking, drinking and physical exercise. This corroborates findings on multiple measures of health from previous research, including studies on mortality [3–8], risk of early retirement from work due to disability [25 [Bibr bibr26-14034948241258674]] and need for long-term antihypertensive medication [26]. The group differences concerning perceived health are of similar magnitude as risk ratios for all-cause mortality between the Swedish-speaking group and the Finnish-speaking group, based on analyses of population register data, or approximately 1.30 and 1.15, respectively [27].

Naturally, the health differences of the language groups were attenuated when covariates were included, but they remained statistically significant. This supports the view that socioeconomic characteristics and health behaviours cannot fully explain the Finnish-speaking/Swedish-speaking health gradient. Belonging to the Swedish-speaking minority in Finland seems to provide some kind of health promoting effect that is hard to grasp in a traditional approach and which might be connected to the Swedish-speaking culture.

The language groups were in this study formed based on response to the question of the respondent’s and the previous generation’s dominating language. It is reasonable to assume that individuals from families who have been Swedish speakers for more than one generation are representative of the Swedish-speaking culture in Finland. Accordingly, it appears correct to assume that the cultural effect would be weaker in individuals whose dominating language is Finnish, but whose parents’ dominating language was Swedish.

These assumptions were supported by the results as the odds ratio for better perceived health were the highest for people in the Swedish-speaking group who were part of families that had spoken the language for at least two generations and the lowest among the corresponding Finnish-speaking group. Consistently, the odds ratios for the mixed language-speaking group lay between these other two groups, although not showing significant differences from any of the groups. These language-group differences are of similar magnitude as risk ratios for all-cause mortality between corresponding groups, based on analyses of population register data [18], and hint toward a potentially important role of culture.

If the health advantage of these three language groups in Finland could be attributed to cultural differences, what could characterize these differences? These kinds of differences can be reflected in health behaviour but without only including the more obvious health impacts but taking also into account the more gradual and indirect cultural consequences. A previous study [12] reviewing national survey data from more than two decades showed that Swedish speakers in Finland smoked less and consumed less alcohol than corresponding Finnish speakers. Additionally, another earlier study [13)] particularly pointed out that quantitatively there were not any great differences in alcohol consumption between the language groups per se, but that the consumption by Finnish speakers was somewhat more concentrated to fewer occasions and thus seemingly more oriented towards intoxication. However, adjusting for alcohol consumption did not remove the statistical significance of the slight advantage related to perceived health among Swedish speakers.

Another earlier national register-based study [15] also pointed at culture as a possible health promoting factor, as it showed that the unemployment rate was lower among Swedish speakers and periods of unemployment were on average shorter compared with corresponding Finnish regions. This ‘accumulation of social capital’ among Swedish speakers in Finland has been proposed as an explanation for their health advantage [25]. However, this idea was not supported in results from the present study, as no significant differences of social integration, assessed in number of close friends [unpublished, data not shown], were detected. From a historical perspective, the Swedish-speaking minority in Finland should be considered being more culturally than genetically determined [28], even though some recent analysis hints towards potential genetic differentials between the populations in the western and eastern regions of Finland [29]. Swedish speakers in Finland live in regions of lower morbidity, which generally might increase their relative health advantage [30], but regional concentration also enhances the possibilities of maintaining an own specific culture, based in part in language use. This idea is somewhat supported by research on the so-called Hispanic paradox. Immigrants from Spanish-speaking countries who live in the US have been shown to have better health outcomes on some health measures, although their socioeconomic status in many areas of the US places them at disadvantages. The ability to maintain strong relationships based on shared language use, despite the wide range of cultures found in Central and South America, does point to language as a key shaper of culture. Llabre [31], in an article discussing why Hispanics in the US may have fewer health problems related to cardiovascular reactivity compared with other groups, cites features of the Spanish language, including the use of the subjunctive to discuss possible outcomes of challenging situations, thereby limiting stress accumulation. The interaction between group density and language should also be explored, particularly as language speakers who have experienced improvements in social status may be isolated from others who speak the same language. Measuring health changes as they relate to generational shifts in language use offers new opportunities to promote better quality of life for communities. To that end, there is also a unique opportunity to compare Swedish speakers in Finland with Finnish speakers in Sweden. A large wave of immigrants from Finland arrived in neighbouring Sweden during the 1960s and 1970s, to work in manufacturing and heavy industry. Studies of this group, whose children all grew up speaking Swedish, could provide better understanding as to the impacts of language shift over a much shorter period – if their children enjoy better health, then this could provide additional evidence that aspects of Swedish culture may confer health protection.

Residents in nations with developed economies are, on average, rather well acquainted with general features of a healthy lifestyle, but nevertheless do not necessarily succeed in changing their health behaviour accordingly. This suggests that getting people motivated to adopt healthy behaviours may lie more in attitudes and emotions, rather than lack of information and knowledge alone. For example, in many cultures, alcohol use is viewed more as something as a drink shared during social interactions rather than a way to reach intoxication. This kind of cultural influence might provide a partial explanation to the present findings, as the relative health advantage of Swedish speakers was robust to adjustment of demographic or health behavioural differences.

Being a member of the Swedish-speaking minority in Finland does not necessarily mean that health by itself gains more attention within this group, but that social circumstances and situations are more health promoting and less health endangering, covering several central domains of health behaviour. The specific features of the Swedish-speaking culture could in future research potentially be studied by in-depth qualitative studies, to better identify and describe them from a health-promoting perspective, and in particular in a comparing perspective toward the Finnish-speaking majority population.

## Strengths

The present study is based on data collected from a large population-based survey. The survey’s respondents answered multiple questions related to health behaviours. The Swedish-speaking minority was over-represented by the double in the respondent group, which thus allowed for more powerful comparison between the language groups. The question of the dominating language of the respondent and his or her parents was a unique characteristic that previously has been utilized only in research based on population-register data.

## Limitations

The study had to be carried out in a cross-sectional manner based on survey data from the year 2012, as the item on the dominating language of the respondent’s present and previous generation was added to this survey. The response rate of the first survey was also moderate, or 40.0%, but it seems not to have generated any health selection that would affect the results of our study [19,20].

## Conclusions

The results gave support to the argument that the health advantage of Swedish speakers in Finland as compared with Finnish speakers may in part be culturally related, and not merely generated by obvious health behavioural mechanisms or socioeconomic differentials. Potentially, the findings can be more generally applied in health promotion, including that of the Finnish-speaking majority population.
